# Primary Rectal Amelanotic Malignant Melanoma: A Rare Case Report

**DOI:** 10.7759/cureus.8115

**Published:** 2020-05-14

**Authors:** Rubab Nafees, Hina Khan, Shahrukh Ahmed, Khursheed Ahmed Samo, Amjad Siraj Memon

**Affiliations:** 1 Surgery, Dr. Ruth K. M. Pfau Civil Hospital Karachi, Karachi, PAK; 2 Surgery, Dow University of Health Sciences, Civil Hospital Karachi, Karachi, PAK

**Keywords:** colorectal cancer, malignant melanoma, amelanotic, anorectal melanoma

## Abstract

Malignant melanoma of the rectum comprises 0.5%-4% of all anorectal cancers. Malignant melanoma of the rectum is exceptionally a rare disease. It commonly affects the fifth or sixth decade, with nonspecific symptoms such as rectal bleeding or anal pain. After skin and retina, anorectum is the third common site for malignant melanoma. Proper diagnosis is difficult in the majority of cases due to lack of pigmentation and amelanotic histological appearance. Prognosis is very poor with a median survival of 24 months and five-year survival of 10%-15%. Anorectal malignant melanomas disseminate along the submucosal planes, therefore complete resection at the time of diagnosis is usually not possible.

## Introduction

Malignant melanoma of the rectum is an uncommon disease, which constitutes 0.5%-4% of all anorectal malignancies and less than 1% of all melanomas [1-3]. It usually produces local symptoms in the fifth or sixth decade of life [1]. After skin and retina, anorectum is the third common site for malignant melanoma. Patients often present with nonspecific complaints such as rectal bleeding or anal pain [2,3]. It affects the Caucasian race commonly. A prompt diagnosis is more difficult as 80% of lesions lack pigmentation and up to 20% of tumours are histologically amelanotic [4,5]. Human melanoma black-45 (HMB-45), soluble 100% (S-100), and melanoma-associated protein A (Melan A) are immunohistochemical stains required for the diagnosis. Prognosis is dismal with a median survival of 24 months and five-year survival of 10%-15% [1,6]. Although surgery is the mainstay of treatment, wide local excision and abdominoperineal resections are the options according to the stage of the disease, but presently, there is no consensus on which surgical approach is favorable [3]. It is resistant to radiotherapy and poorly responsive to chemotherapy as well. Anorectal malignant melanomas spread along submucosal planes, therefore, complete resection is impossible at the time of diagnosis, therefore, almost all patients die because of metastases [7,8].

## Case presentation

A 69-year-old male, with no known comorbidities presented with complains of tenesmus and bleeding per rectum for the last four months. There was no documented weight loss or fever. On digital rectal examination, an irregular thickening of the posterior anorectal wall was noted, starting from the anal verge at 6 o’clock position, and extending upwards with an upper limit not reachable; the anterior anorectal wall was normal. The finger was blood stained. The rest of the systemic examination was unremarkable.

Colonoscopy showed eccentric, ulcerated, friable growth in the rectum starting from the anal verge extending up to 16 cm. There was no luminal narrowing. A biopsy was taken.

CT scan of the chest abdomen pelvis (CAP) showed large polypoidal mass involving proximal and distal rectum, laterally infiltrating the right levator ani muscle, and superiorly reaching up to S2 vertebra, sparing the sigmoid colon. There was significant perirectal fat stranding with lymphadenopathy. No pleural or pulmonary metastasis.

MRI pelvis showed irregular, circumferential, polypoidal abnormal intensity mass lesion involving the anorectal canal. The mass extended from the anal verge, proximally till the distal sigmoid colon. The maximum craniocaudal length of mass measured 15.0 cm, transverse dimension measured 5.2 cm, maximum single wall thickness measured 4.4 cm. Marked perirectal fat stranding was seen extending posteriorly up to the presacral space. Bilateral levator ani muscles were involved. Anteriorly, there was a loss of fat planes with the prostate. There were enlarged perirectal lymph nodes; the largest one measured 1.5 x 1.1 cm.

carcinoembryonic antigen (CEA) level was 1.7. Histopathology report showed largely necrotic tissue; few atypical viable cells were present which were positive for S-100 and melanocyte marker HMB-45. A possibility of malignant melanoma (amelanotic) was raised.

**Figure 1:**
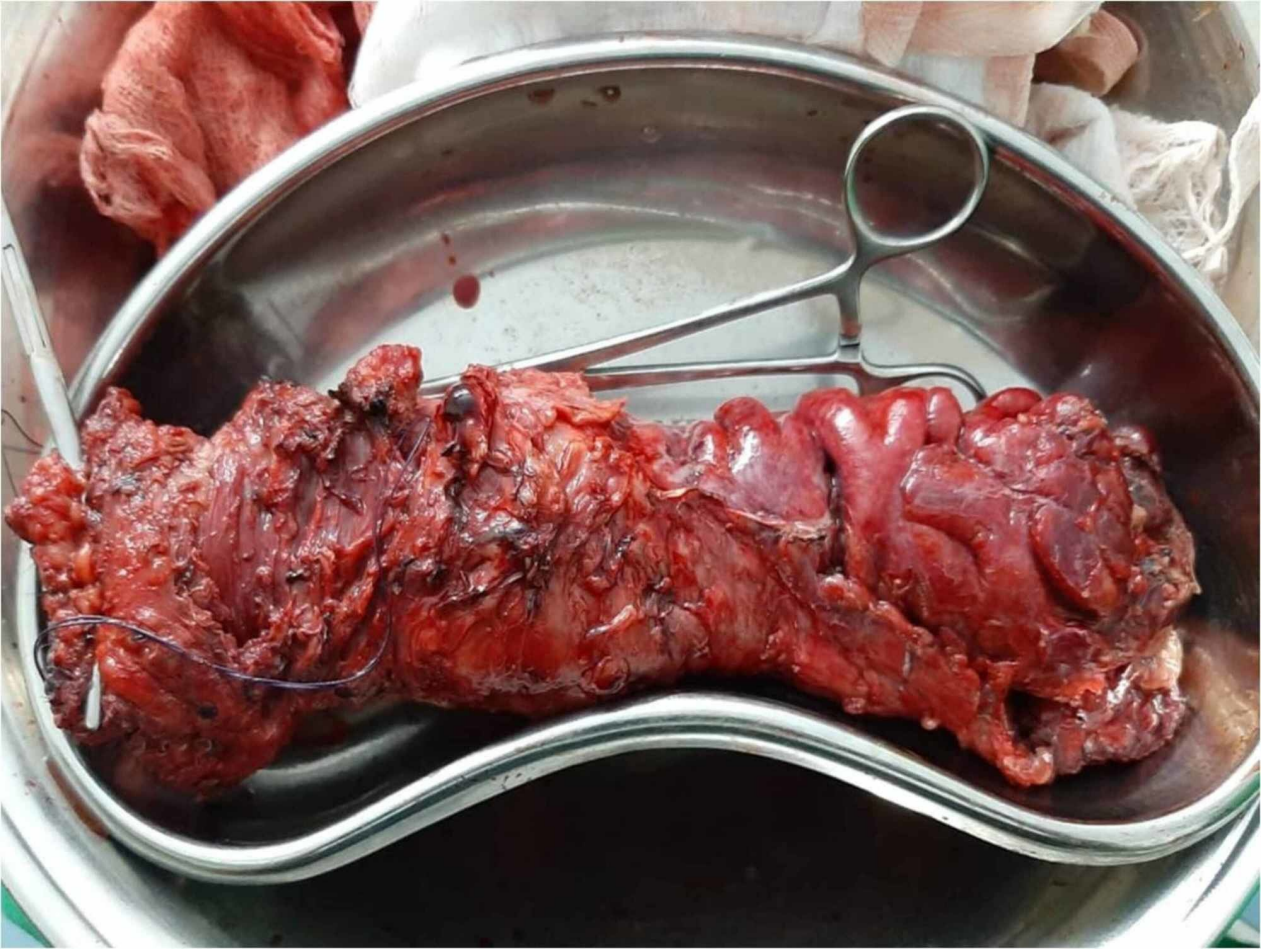
Postoperative specimen of anorectal malignant melanoma

Oncology opinion was taken and the patient was planned for abdominoperineal excision of rectum (APER). Operative findings revealed the tumor involving anorectum up to the distal sigmoid colon. Tumour was adherent to the prostate. No liver or peritoneal metastasis was found. Figure 1 shows the postoperative specimen. Postoperative recovery was uneventful and the patient was discharged home.

Histopathology of the specimen showed tumour infiltrating into the muscularis propria and adipose tissue. The size was 8 x 7 x 6 cm, the thickness was 6 cm, mucosal ulceration was present, both resection margins were tumour free; a total of 17 lymph nodes were recovered out of which two were involved by the tumour, and an extra nodal extension was present. Features were consistent with malignant melanoma.

After six months, the patient presented with complaints of nodular swelling over the perineal scar tissue. Examination under anesthesia was done which showed a 3 x 3 cm lump at the perineal scar site. Wide local excision was done and the sample was sent for histopathology, which showed features of recurrent malignant melanoma; the size of the lesion was 2.5 x 2.5 x 2.2 cm, the closest peripheral margin was 0.4 cm away, and the deep margin was 0.9 cm away. CT scan was unremarkable for distant disease. The patient died seven months after the resection due to metastasis.

## Discussion

Melanocytes are derived from the embryological neural crest and migrate to primarily the skin and other locations. Therefore, cutaneous melanomas account for more than 90% of all melanomas, 5% retinal melanoma, 2% melanoma of unknown origin, and 1% mucosal melanoma [2]. Signs of symptoms are less common in amelanotic malignant melanoma (AMM). Patients who are diagnosed with AMM, already have loco regional metastasis [8]. Lung, liver, brain, bone, and breast are the common sites for distant metastasis [9].

A sigmoido-colonoscopy and endoscopic endorectal ultrasound may be considered to evaluate and diagnose the disease status along with CT scan and MRI pelvis [7].

Immunohistochemical analysis is the cornerstone for the diagnosis of AMM. Anti-S-100 protein is a commonly used stain for AMM and it is highly sensitive for melanocytic differentiation. Other stains are HMB-45, vimentin, and Melan A for the diagnosis of malignant melanoma. In our case, S-100 and melanocyte marker HMB-45 melanin was a diagnostic marker.

Surgery in combination with adjuvant immunotherapy, like interferon, was reported to be a choice of treatment for amelanotic anorectal melanoma if the tumour is localized [10]. Bullard et al. reported that AMM recurrence is not associated with the initial surgical procedure [11]. In our case, we performed abdominoperineal resection. Local excision is preferred by some surgeons, as abdominoperineal resection has a worse prognosis postoperatively [12]. Chemotherapy and radiotherapy have a limited role [13]. Dacarbazine shows a 20% partial response in four to six months [7]. Japan has shown success in immunotherapy recently by using nivolumab (Opdivo), an antibody to programmed death (PD-1) for controlling metastasis [14]. Our patient died after six months of surgery due to distant metastasis like the other case in the literature [15,16].

## Conclusions

Malignant melanoma of the rectum is extremely rare, highly aggressive, and difficult to diagnose. Although surgery remains the cornerstone of treatment, the exact procedure remains controversial. The role of adjuvant therapies is minimal. Survival can be prolonged only by early diagnosis.
